# IL-17A promotes the progression of Alzheimer’s disease in APP/PS1 mice

**DOI:** 10.1186/s12979-023-00397-x

**Published:** 2023-12-14

**Authors:** Min Cao, Jing Liu, Xiaomin Zhang, Yaqi Wang, Yuli Hou, Qiao Song, Yuting Cui, Yue Zhao, Peichang Wang

**Affiliations:** 1 Department of Clinical Laboratory, Xuanwu Hospital Capital Medical University, Beijing, 100053 People’s Republic of China; 2 Department of Clinical Laboratory, Beijing Huairou Hospital, Beijing, 101400 People’s Republic of China

**Keywords:** Alzheimer’s disease, IL-17A, Neuroinflammation, TNFα

## Abstract

**Background:**

Alzheimer’s disease (AD), which is the most common cause of dementia in elderly individuals, is a progressive neurodegenerative disorder. Neuroinflammation, which is an immune response that is activated by glial cells in the central nervous system, plays an important role in neurodegenerative diseases. Many studies have shown that interleukin-17A (IL-17A) plays an important role in AD, but research on the pathological effects of IL-17A on AD is limited.

**Methods:**

We report the effect of IL-17A on AD progression in APPswe/PS1dE9 (APP/PS1) mice, which are the most widely used AD model mice. The BV2 cell line, which is a microglial cell line derived from C57/BL6 mice, was used to establish a cell model to verify the role of IL-17A in neuroinflammation at the cellular level. The HT22 hippocampal neuronal cell line was used to investigate the relationship between IL-17A and Aβ deposition.

**Results:**

In this research, we found that IL-17A promotes the progression of AD in the APP/PS1 mouse model. The role of IL-17A in neuroinflammation is related to tumour necrosis factor (TNF)-α. Circulating IL-17A stimulates the secretion of TNF-α by microglia through the Toll-like receptor 4 (TLR4)/nuclear factor (NF)-κB signalling pathway, thus exacerbating neuroinflammation. In addition, intraperitoneal injection of IL-17A antibody (IL17Ab) significantly improved the cognitive function of APP/PS1 mice.

**Conclusions:**

IL-17A increased TNF-α levels in the brain and exacerbated neuroinflammation through the TLR4/NF-κB signalling pathway and microglial activation in APP/PS1 mice. Moreover, IL-17A promoted the progression of AD by enhancing neuroinflammation, inhibiting microglial phagocytosis, and promoting the deposition of β-amyloid 42 in AD model mice.

**Supplementary Information:**

The online version contains supplementary material available at 10.1186/s12979-023-00397-x.

## Background

Alzheimer’s disease (AD) is a neurodegenerative disease, and its main clinical symptom is neurocognitive decline. AD is caused by damage to neurons in the brain, which are mainly responsible for memory, language, and thinking. As a result, the first symptoms of AD tend to include problems with memory, language, and thinking [1]. The pathology of AD involves the formation of Aβ plaques and neurofibrillary tangles, and the brains of AD patients are easily distinguished from the brains of healthy controls. Moreover, multiple studies suggest the presence of chronic neuroinflammatory responses, which are manifested by reactive microgliosis astrogliosis and elevated levels of proinflammatory cytokines [2].

Microglia, which are the innate immune cells of the central nervous system (CNS), play crucial roles in tissue maintenance, injury response, and pathogen defence in the CNS [3]. With the accumulation of Aβ plaques, tau pathology increases in stressed or damaged neurons, and microglia transform into an inflammatory state; proinflammatory microglia engulf synapses, secrete neurotoxic cytokines that injure neurons, and support spread of tau pathology [4]. Although the main pathological features of AD include the formation of intracellular neurofibrillary tangles and the deposition of extracellular neurotoxic plaques that are primarily composed of Aβ, neuroinflammation has also been observed in AD; this neuroinflammation may be the cause of the disease or a possible reaction to pathology.

Interleukin-17A (IL-17A) is a proinflammatory cytokine. Th17 cells, macrophages, γδT cells, cytotoxic T cells, and innate lymphoid cells are the main sources of IL-17A [5]. The primary functions of IL-17A include the induction of chemokine, IL-6, and granulocyte colony-stimulating factor expression [6]. Additionally, Th17 cells are well-known targets in the treatment of psoriasis. A phase 2 clinical trial showed the effectiveness of treatment that inhibited IL-17A, which indicates the pathological role of IL-17A in mediating important inflammatory pathways in psoriasis [7]. In addition to its role in psoriasis, most lines of evidence suggest a pathological role of IL-17A in neurodegenerative diseases that affect the CNS.

IL-17A may play a significant role in the pathogenesis of AD. Researchers have observed an increased number of γδT cells, which are the major IL-17-producing cells, in the brains and meninges of the 3xTg-AD model [8]. Th17 cells promote local immunoamplification loops and blood–brain barrier (BBB) breakdown in the meninges [9]. Moreover, studies have revealed elevated levels of IL-17A in the plasma and cerebrospinal fluid (CSF) of AD patients [10]. These results raise the possibility that IL-17A plays an important role during AD.

Here, we aimed to investigate the mechanism by which IL-17A contributes to AD and the effect of IL-17A inhibition on the cognitive function of AD model mice.

## Methods

### Human samples

For this study, 29 individuals with mild cognitive impairment from Alzheimer’s disease (MCI), and 48 individuals with dementia of Alzheimer type (DAT) from the Xuan Wu Hospital in Beijing were included. Subjects were diagnosed with DAT according to the National Institute on Aging and Alzheimer’s Association diagnostic criteria. For MCI, the diagnosis was made according to the neurological diagnosis [11], with a Clinical Dementia Rating scale score of 0.5. Meanwhile other nervous system diseases (stroke, Parkinson’s disease, etc.) and psychiatric illnesses (schizophrenia, major depressive disorder, etc.) were excluded. In addition, 20 Parkinson’s disease (PD) patients were selected according to the Movement Disorder Society Task Force criteria [12], and 15 patients with vascular dementia (VaD) were diagnosed by NINDS-AIREN criteria [13]. Finally, age- and sex-matched healthy controls (HC) were selected from the health examination. Mini-Mental State Examination (MMSE) and Montreal Cognitive Assessment (MoCA) scores were performed on all subjects to assess cognitive status. Blood samples were collected after fasting. Whole blood was centrifuged at 3000 × g for 5 min at room temperature. Then, the serum was immediately transferred to storage tubes marked with the name and sex of the participant and immediately frozen in a − 80 °C freezer. All the subjects were approved by the ethics committee of Xuanwu Hospital of Capital Medical University (Beijing, China), and written informed consent was obtained from all the participants or their guardians.

### Mice

The animal study protocol was approved by the Bioethics Committee of Beijing Huayuanshidai Technology Co., LTD. The research was conducted in accordance with the Guide for the Care and Use of Laboratory Animals. 2-, 5-, and 9-month-old male APP/PS1 transgenic mice as well as age- and sex-matched male wild-type (WT) mice were obtained from Beijing Huafukang Biotechnology Company (Beijing, China).

### In vivo treatments

Intraperitoneal injection: To test the effect of IL-17A, we randomly divided the 8-month-old APP/PS1 mice into four groups: (1) APP/PS1 mice that were intraperitoneally injected with recombinant IL-17A (0.05 mg/kg, PeproTech, USA); (2) APP/PS1 mice that were intraperitoneally injected with vehicle (phosphate-buffered saline (PBS)); (3) APP/PS1 mice that were intraperitoneally injected with IL-17A antibody (IL17Ab) (5 mg/kg, BioLegend, USA); and (4) APP/PS1 mice that were intraperitoneally injected with isotype control (5 mg/kg, BioLegend, USA) twice a week for 4 weeks.

### Behavioural studies

Morris water maze (MWM): Spatial memory performance was analysed using the MWM test as previously described. In brief, all mice were trained in the MWM for 5 days. The water maze was covered with milk and kept at 21 °C. A platform was hidden 1 cm below the surface of the opaque water. The pool was divided into four equal quadrants. On the training days, the mice were placed in each quadrants and trained to find the platform. The escape latency was measured in each trial. After the initial four days training, followed by the probe trial on the fifth day. The mice were placed in the pool without the platform, the number of times the mice crossed the previous location of the platform as well as the time spent in the quadrant that previously contained the platform were recorded for 60 s.

### Immunofluorescence

After anaesthesia, the mice were perfused with 4% paraformaldehyde (PFA), and brain tissues were isolated. Then, the tissues were fixed overnight in 4% PFA solution at 4 °C. The brains were cut into 10 µm sections using a cryostat microtome. The sections were washed with PBS, blocked with 10% donkey serum for 30 min, and then incubated overnight at 4 °C with a primary antibody against the microglial cell marker IBA‐1 (1:1000). On the second day, the sections were washed and incubated for 1 h with the secondary antibody solution (donkey antirabbit IgG Alexa Fluor 488; 1:1000). The nuclei were counterstained with 4′,6-diamidino-2-phenylindole (DAPI) (1:1000). The sections were washed with PBS and coverslipped with mounting medium, and images were obtained using an automated fluorescence microscope.

### Thioflavin-S staining

The sections were first washed with TBS to minimize background staining and then incubated with 0.3% thioflavin-S dissolved in 50% ethanol for 8 min. This step was followed by washing with 50% ethanol for 5 min and incubation in TBS for 5 min.

## Cells

Murine microglia (BV2 cells) were obtained from the National Infrastructure of Cell Line Resource (Beijing, China). The BV2 cells were maintained in Roswell Park Memorial Institute (RPMI) 1640 supplemented with 10% fetal bovine serum (FBS) at 37 °C in 5% CO_2_. The HT22 hippocampal neuronal cell line was obtained from LMAI Bio (Shanghai, China). The HT22 cells were maintained in Dulbecco’s modified Eagle’s medium (DMEM) supplemented with 10% FBS at 37 °C in 5% CO_2_.

### Microglial phagocytosis assay

BV2 cells were cultured in 24-well plates (2.5 × 10^4^ cells/well). To analyse the effect of IL-17A on the phagocytosis of BV2 cells, we divided these cells into four groups, namely, (1) control BV2 cells, (2) Aβ-stimulated BV2 cells, (3) IL-17A–stimulated BV2 cells, and (4) Aβ-stimulated BV2 cells treated with IL-17A, as previously described with slight modifications [14]. In brief, latex beads were incubated with FBS for 1 h at 37 °C and then diluted to 0.01% in DMEM. After the treatment of BV2 cells with Aβ with/without IL-17A, the BV2 cell culture medium was replaced with RPMI 1640 medium containing beads, and the cultures were incubated at 37 °C for 1 h. The cultures were thoroughly washed with ice-cold PBS five times. Then, the cells were fixed using 4% PFA for 15 min. Finally, the cytoskeleton was labelled with ActinRed (1:50). The BV2 cells were observed under a fluorescence microscope, and their phagocytic efficiency was calculated.

### Western blotting (WB)

Tissues and cellular proteins were lysed in radio immunoprecipitation assay (RIPA) lysis buffer (Solarbio, Beijing, China). The protein concentration was determined using a BCA Protein Assay kit (Thermo Fisher Scientific, Massachusetts, United States). Proteins were separated by sodium dodecyl sulfate‒polyacrylamide gel electrophoresis (SDS‒PAGE) and then transferred to polyvinylidene difluoride (PVDF) membranes. The membranes were blocked with 5% milk for 1 h and then immunoblotted with primary antibodies against Toll-like receptor 4 (TLR4) (1:800 dilution; Abcam), MyD88 (1:800 dilution; Abcam), nuclear factor (NF)-κB (1:800 dilution; Abcam), APP (1:1000 dilution; Abcam), and β-actin (1:5000 dilution; Abcam) at 4 °C overnight. Then, the membranes were incubated with a horseradish peroxidase (HRP)-conjugated secondary antibody at room temperature for 1 h and visualized using enhanced chemiluminescence.

### Reverse transcription-quantitative polymerase chain reaction (RT‒qPCR)

Total RNA was isolated from tissues and cells with TRIzol® reagent. Reverse transcription was performed using cDNA synthesis kits in accordance with the manufacturer’s instructions (Abm, USA). RT‒qPCR was performed using SYBR (Takara, Japan) on a Roche 480 machine (Basel, Switzerland). Table 1. shows the sequences of the primers that were used in the study.

**Table 1:** Sequences used for RT‒qPCR

**Target gene**	**Forward primer**	**Reverse primer**
TNF-α	GAACTGGCAGAAGAGGCACTC	AGGGTCTGGGCCATAGAACTG
IL17A	TCATCCCTCAAAGCTCAGCG	TTCATTGCGGTGGAGAGTCC
TLR4	AGCTCCTGACCTTGGTCTTG	CGCAGGGGAACTCAATGAGG
NF-κB	CTGGGCACCAGTTCGATGG	GACAGCATAAGGCACACACTT
MYD88	TCATGTTCTCCATACCCTTGGT	AAACTGCGAGTGGGGTCAG
IL-1β	AATGATCTGTTCTTTGAGGCTGAC	CGAGATGCTGCTGTGAGATTTGAAG
IL-4	TCACTGACGGCACAGAGCTA	CCTTCTCCTGTGACCTCGTT
IL-10	AAGGGTTACTTGGGTTGCCA	TTTCTGGGCCATGCTTCTCT

### Plasmid construction and transfection

The APP gene sequence (mouse, NM_001198823) was searched in the National Center for Biotechnology Information website database. Based on the target gene sequence, the fragment was amplified and inserted into the PHS-AVC-LW1841 vector. We transfected the APP recombinant plasmid into HT22 cells using jetOPTIMUS (Polyplus, France) for 24 h. After transfection, the cells were harvested and used for protein analysis.

### Enzyme-linked immunosorbent assay (ELISA)

The serum concentration of IL-17A, the brain extract, cell culture medium and cell lysate concentration of Aβ_1-40_ and Aβ_1-42_ and the cell culture medium concentration of TNF-α were measured using ELISA kits (MLbio, Shanghai, China) according to the manufacturer’s instructions. All the samples were measured in duplicate, and mean CV was less than 5%.

### Statistical analysis

The date is presented as the mean ± standard deviation. The results are analyzed using GraphPad Prism software by the independent-samples t-test or One-way analysis. For determination of the correlation, linear-regression analysis was used. Images were assessed using the ImageJ software. *P* < 0.05 was considered significant.

## Results

### Changes in inflammatory factors in AD

**Fig. 1:**
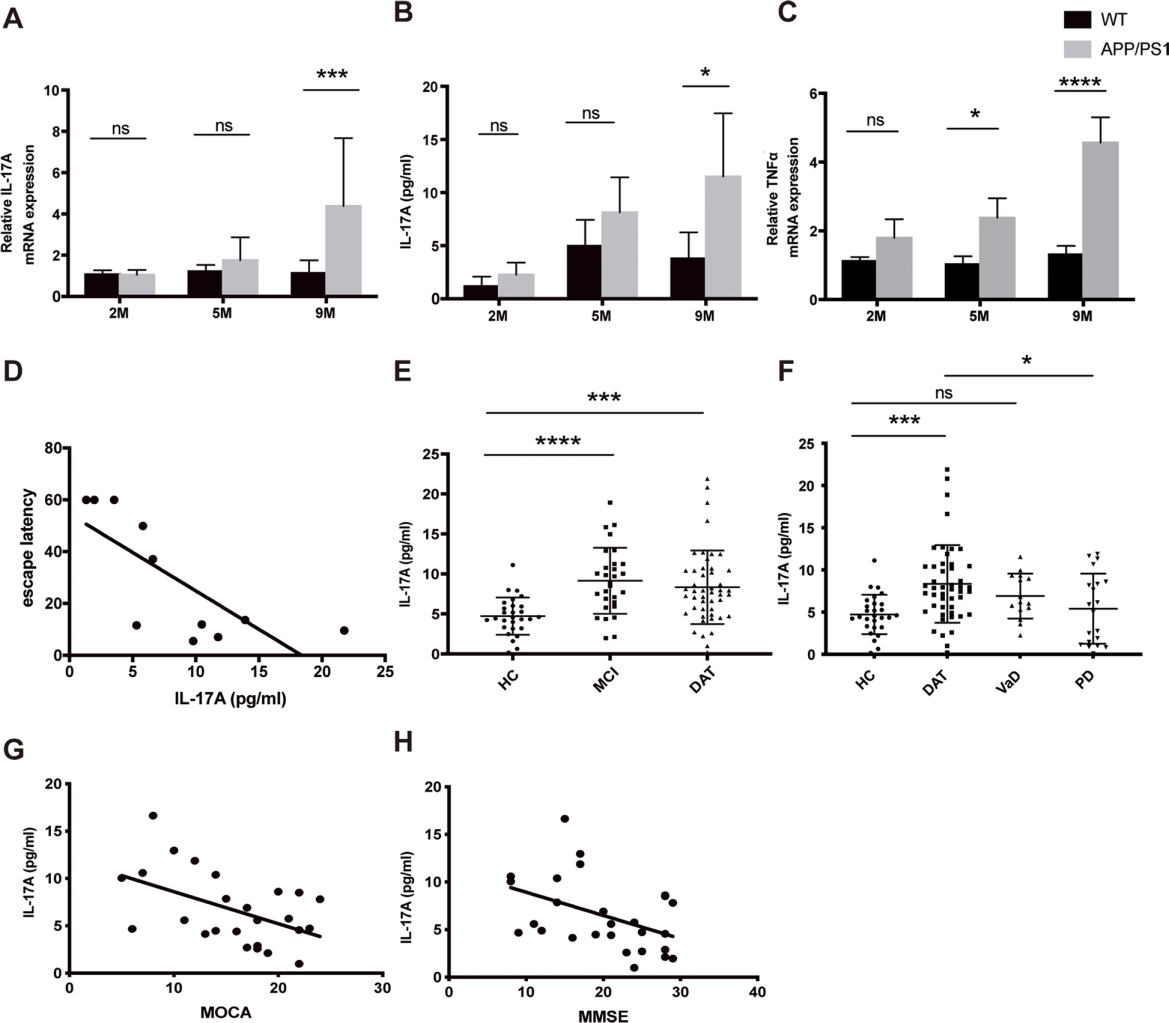
Level of IL-17A in samples from model mice and AD patients with different stages of disease. **A** mRNA levels of IL-17A in the hippocampus of 2-, 5-, and 9-month-old APP/PS1 mice. *n* = 5 for each group. **B** Concentrations of serum IL-17A levels in 2-, 5-, and 9-month-old APP/PS1 mice. *n* = 5 for each group. **C** mRNA levels of TNF-α in the hippocampus of 2-, 5-, and 9-month-old APP/PS1 mice. *n* = 5 for each group. **D** Relationship of serum IL-17A concentration with escape latency in 9-month-old APP/PS1 mice. **E** Serum IL-17A levels in AD patients with different stages of disease. **F** Serum IL-17A levels in patients with AD and other neurodegenerative diseases. **G** Relationship of the serum IL-17A level with the MoCA score of AD patients according to linear regression analysis. **H** Relationship of the serum IL-17A level with the MMSE score of AD patients according to linear regression analysis. One-way analysis of variance was used to assess the differences among more than two groups. The values are expressed as the mean ± standard deviation (****P < 0.0001, ***P < 0.001, **P < 0.01, and *P < 0.05). HC: healthy controls; MCI: mild cognitive impairment; DAT: dementia of Alzheimer type; VaD: vascular dementia; PD: Parkinson’s disease. The data are representative of at least three independent experiments

RT‒qPCR was used to analyse the levels of proinflammatory and anti-inflammatory factors in the hippocampus of APP/PS1 mice of different ages (in months) and age-matched WT mice (Supplemental Fig. 1). The mRNA level of IL-17A in the brain increased with age (Fig. 1A). ELISA was used to measure the IL-17A level in the peripheral blood of mice of different ages, and the results were consistent with those observed in the brain (Fig. 1B). Moreover, the mRNA level of TNF-α increased with age, which were consistent with those of previous studies (Fig. 1C). To further explore the effects of IL-17A on cognitive function, cognitive function was assessed using the MWM. We observed that serum IL-17A levels were significantly correlated with escape latency (Fig. 1D).

### IL-17A in AD patients at different stages

Blood samples were collected from patients with neurodegenerative diseases (including AD, PD, and VaD), and sex- and age-matched HC were recruited from among physical examination patients. Figure 1E shows the concentrations of IL-17A in the samples from AD patients with different degrees of disease progression. The detectable levels of IL-17A were significantly higher in AD patients than in the controls. Moreover, the serum levels of IL-17A in PD and VaD patients were also higher than those in the controls (Fig. 1F). Linear regression analysis showed that the serum IL-17A level was significantly correlated with the MMSE and MoCA scores of AD patients (Fig. 1G-H). These results indicate that IL-17A may play a role in the progression of AD and is associated with cognitive function.

### IL-17A exacerbated cognitive impairment in APP/PS1 mice

**Fig. 2:**
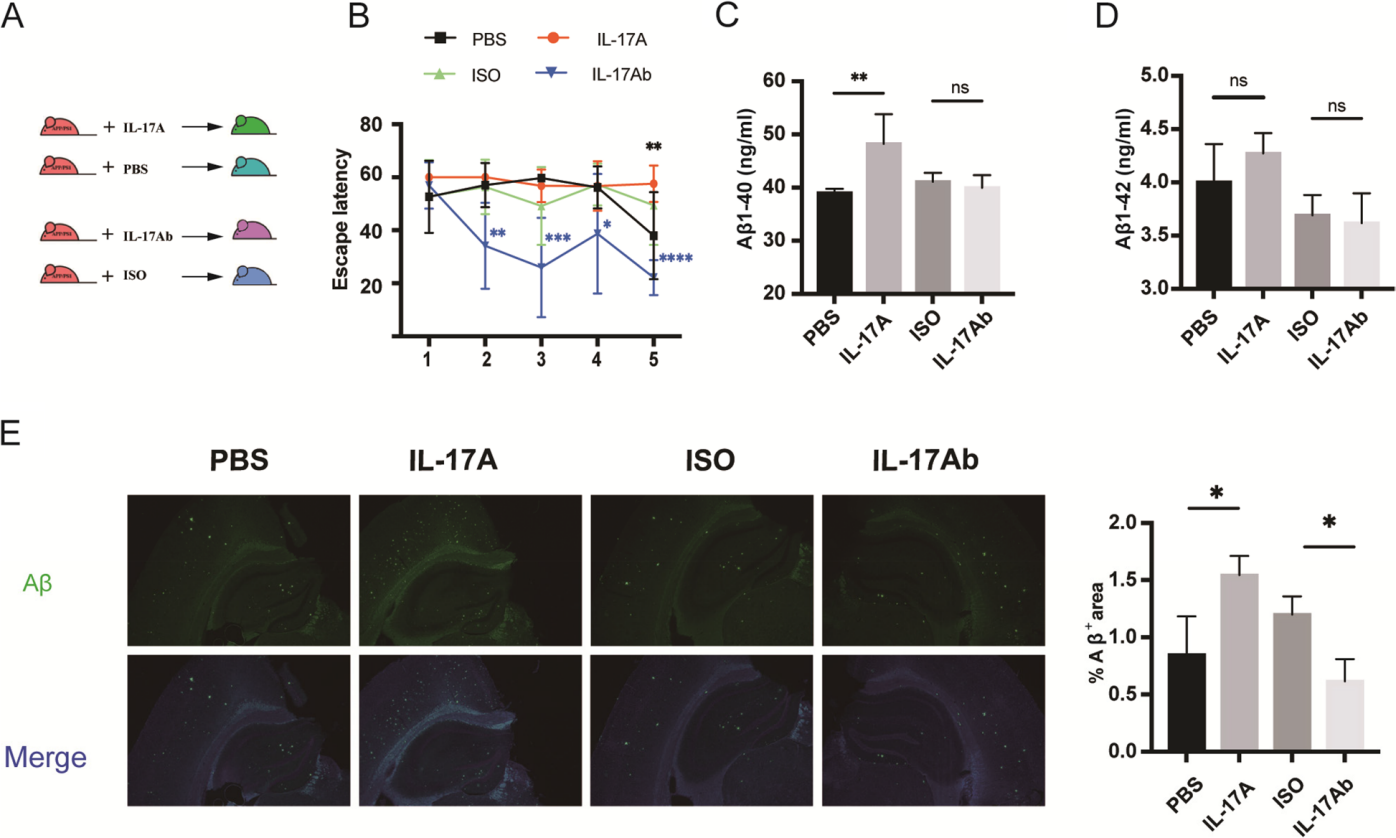
IL-17Ab ameliorated the decrease in spatial and working memory in APP/PS1 mice. **A** Schematic of IL-17A or IL-17Ab administration to APP/PS1 mice via intraperitoneal injection. **B** Escape latency of APP/PS1 mice injected with IL-17A or IL-17Ab during the training phase of the MWM test. *n* = 5 for each group. **C** Aβ_1-40_ levels in the brain tissues of APP/PS1 mice after the injection of IL-17A or IL-17Ab. **D** Aβ_1-42_ levels in the brain tissues of APP/PS1 mice after the injection of IL-17A or IL-17Ab. **E** Representative immunofluorescence images of Aβ plaques in APP/PS1 mice after the injection of IL-17A or IL-17Ab. The values are expressed as the mean ± standard deviation (****P < 0.0001, ***P < 0.001, **P < 0.01, and *P < 0.05). The data are representative of at least three independent experiments

Based on the above studies, the levels of IL-17A increased with the age of APP/PS1 mice. To further explore the effects of IL-17A on cognitive function, we intraperitoneally injected APP/PS1 mice with recombinant IL-17A or anti-IL-17A using PBS and isotype (ISO) as controls (Fig. 2A). Cognitive ability was assessed by the MWM test. As shown by the MWM tests, compared with the injection of PBS, injection of IL-17A significantly decreased cognitive function of the APP/PS1 mice, and their escape latency significantly increased on day 5. Compared with the injection of ISO, the injection of anti-IL-17A substantially improved the cognitive function of APP/PS1 mice. The escape latency of the APP/PS1 mice was markedly reduced from day 2 to day 5 (Fig. 2B). Compared with the PBS injection, the Aβ_1-40_ in the brain tissue of mice considerably increased after the injection of recombinant IL-17A, and Aβ_1-42_ showed no sizable change (Fig. 2C–D). Subsequently, we performed thioflavin-S staining to examine senile plaque deposition in the brain tissues of the mice. After the intraperitoneal injection of recombinant IL-17A, 9-month-old APP/PS1 mice exhibited an increase in senile plaque deposition in brain tissues, which was effectively reduced by anti-IL-17A antibody treatment (Fig. 2E). Altogether, these data suggest that IL-17A substantially accelerates Aβ plaque deposition in the brain tissue of APP/PS1 mice and that anti-IL-17A treatment results in a decrease in Aβ plaque deposition.

### IL-17A elevated the level of inflammation via the TLR4/NF-κB signalling pathway and microglial activation in the brains of APP/PS1 mice

The above studies showed that after the injection of IL-17A in vitro, the cognitive function of APP/PS1 mice decreased, the escape latency was prolonged, and Aβ plaques were deposited in the brain. However, the IL-17A antibody can considerably improve the cognitive function of APP/PS1 mice. We speculate that IL-17A increases neuroinflammation by changing the levels of inflammatory factors in the brain, thus exacerbating cognitive decline. We observed that the trend of change in TNF-α in the brains of APP/PS1 mice was consistent with that of IL-17A. Thus, we speculated that IL-17A injection in vitro can increase the level of TNF-α in the brain.

**Fig. 3:**
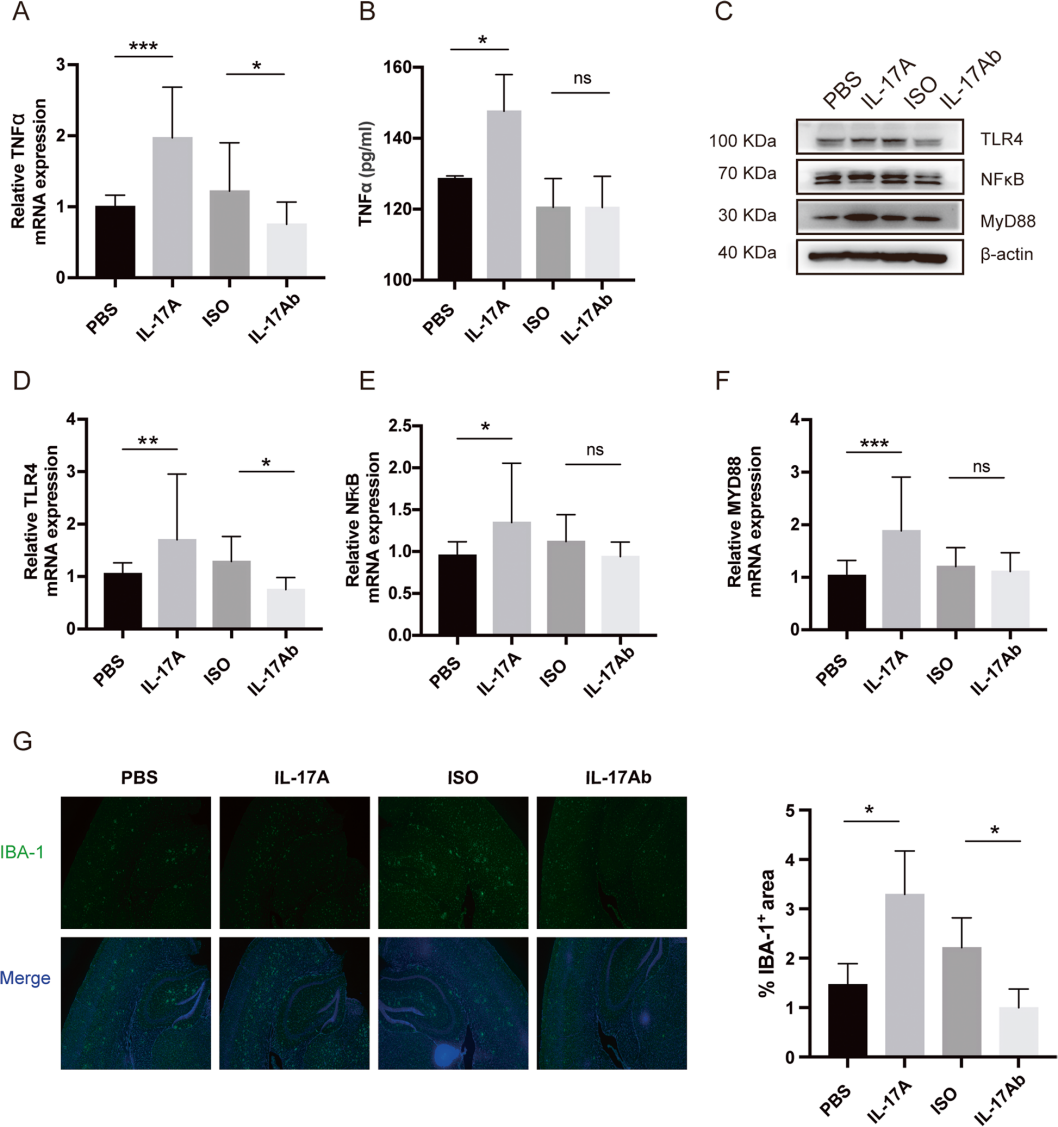
IL-17A elevated the level of inflammation through the TLR4/NF-κB signalling pathway and microglial activation. **A** TNF-α mRNA levels in the brain tissue of APP/PS1 mice after injection of IL-17A or IL-17Ab were determined by RT‒qPCR. **B** TNF-α levels in the brain tissue of APP/PS1 mice after the injection of IL-17A or IL-17Ab were determined by ELISA. **C** Expression levels of TLR4/NF-κB signalling pathway-related proteins in the brain tissues of APP/PS1 mice that were injected with IL-17A or IL-17Ab. **D**–**F** mRNA expression levels of TLR4, NF-κB, and MYD88 in the brain tissue of APP/PS1 mice after the injection of IL-17A or IL-17Ab. **G** Representative immunofluorescence images of IBA-1^+^ microglia in APP/PS1 mice after the injection of IL-17A or IL-17Ab. The values are expressed as the mean ± standard deviation (****P < 0.0001, ***P < 0.001, **P < 0.01, and *P < 0.05). The data are representative of at least three independent experiments

The hippocampal tissues of the above model mice were collected, and the TNF-α levels in the hippocampi were measured by RT‒qPCR and ELISA. The results showed that the mRNA expression of TNF-α in the brain tissue substantially increased after the injection of recombinant IL-17A, and this expression was effectively reduced by anti-IL-17A treatment compared with ISO (Fig. 3A). TNF-α levels were measured by ELISA and found to be consistent with the mRNA levels (Fig. 3B). These results indicate that IL-17A may affect the progression of AD by regulating TNF-α.

Microglia express a variety of receptors, including TLRs, which are pattern recognition receptors [15]. When microglia are stimulated by debris from apoptotic and necrotic cells and heat shock proteins, TLR4 is activated, which further stimulates the downstream transcription factor NF-κB and promotes the secretion of the proinflammatory cytokines TNF-α, IL-1β, and IL-6 [16]. IL-17A interacts toward specific receptors (IL-17RA and IL17RC). This interaction leads to a cascade of intracellular signaling pathways that induces direct and indirect activation of the NF-κB signaling pathway. The expression levels of IL-17RA, IL17RC and key proteins in the TLR4/NF-κB signalling pathway in the brains of the model mice were measured. Considering the activation of TLR4, the expression of CD80, CD86 and MHCII were also detected. WB data revealed that the APP/PS1 mice that were treated with recombinant IL-17A exhibited increases in the expression of TLR4, MYD88, NF-κB, IL-17RA, IL17RC, CD80, CD86 and MHCII compared with the control mice. Anti-IL-17A antibody treatment caused the downregulation of TLR4, MYD88, NF-κB, IL-17RA, IL17RC, CD80, CD86 and MHCII in the hippocampal tissues of APP/PS1 mice (Fig. 3C, Fig. S2). The mRNA levels were consistent with the protein levels (Fig. 3D–F). These results suggest that IL-17A may enhance TNF-α levels in the brain by activating the TLR4/NF-κB signalling pathway.

Microglial activation is a part of the immune response of the human brain [17]. We used IBA-1 as a specific marker to analyse the changes the microglia of the cortex and hippocampus of APP/PS1 mice that were treated (8 months) with recombinant IL-17A or an anti-IL-17A antibody. In contrast to the activated microglia (amoeboid) that were observed in APP/PS1 mice that were treated with PBS, increased microglial accumulation was observed in the cortex and hippocampus of APP/PS1 mice that were treated with recombinant IL-17A. However, the number of IBA-1-positive microglia markedly decreased after the injection of the anti-IL-17A antibody (Fig. 3G). Thus, our results indicate that IL-17A activates microglia, which secrete inflammatory mediators (TNF-α) to induce neurotoxicity.

### IL-17A upregulated TNF-α through the TLR4/NF-κB signalling pathway in BV2 cells

**Fig. 4:**
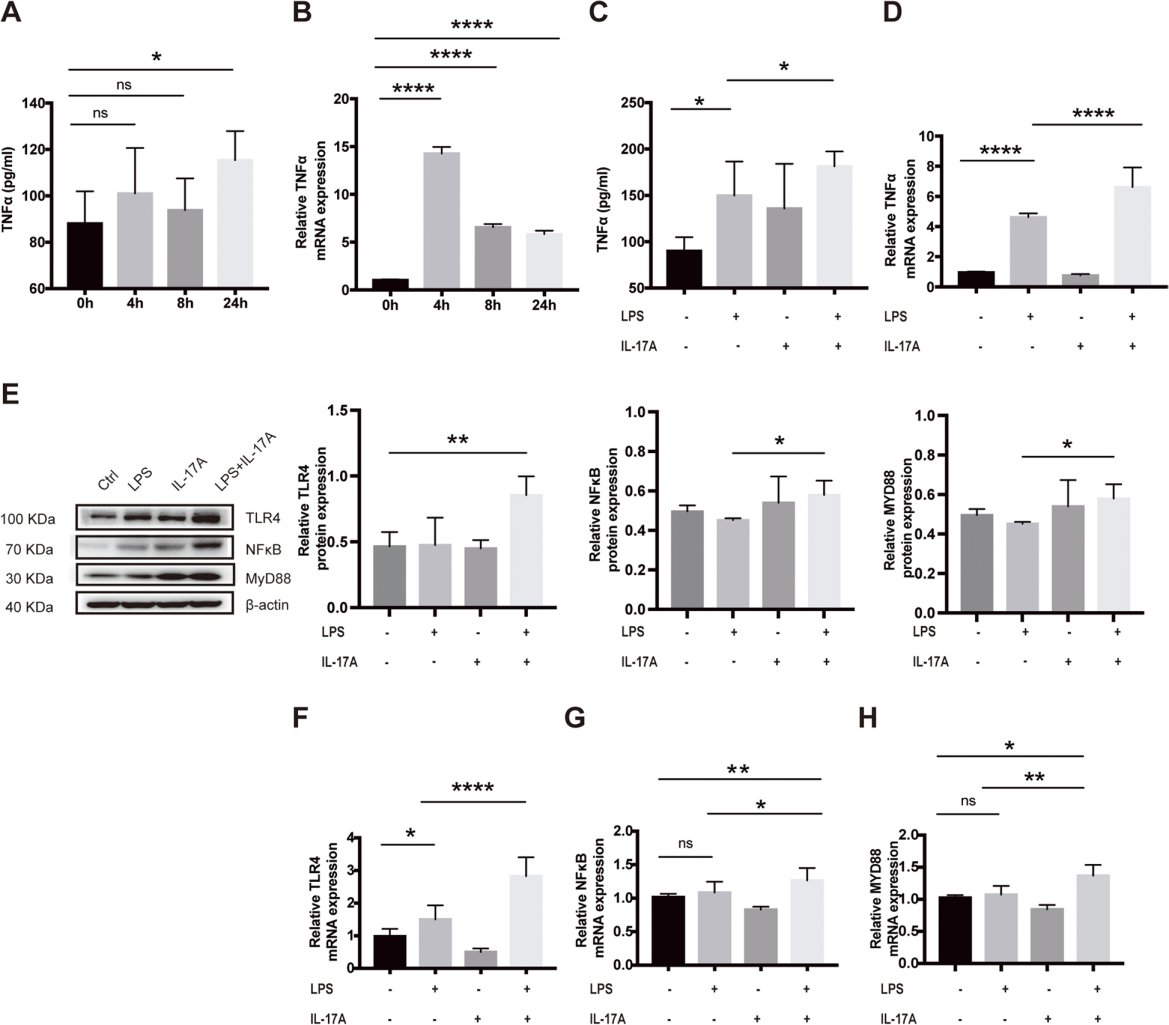
IL-17A upregulated TNF-α through the TLR4/NF-κB signalling pathway in BV2 cells. **A** Changes in the TNF-α levels the supernatants of BV2 cells that were stimulated with LPS at different times (ELISA). **B** Changes in TNF-α mRNA expression in BV2 cells that were incubated with LPS at different times (RT‒qPCR). **C** TNF-α levels in the supernatants of BV2 cells that were incubated with LPS and IL-17A for 24 h. **D** TNF-α mRNA levels in BV2 cells that were incubated with LPS and IL-17A for 4 h. **E** Expression levels of TLR4/NF-κB signalling pathway-related proteins in BV2 cells after IL-17A stimulation for 24 h under inflammatory conditions. **F**–**H** mRNA expression levels of TLR4, NF-κB, and MYD88 in BV2 cells that were stimulated with IL-17A for 24 h under inflammatory conditions. The values are expressed as the mean ± standard deviation (****P < 0.0001, ***P < 0.001, **P < 0.01, and *P < 0.05). The data are representative of at least three independent experiments

By establishing an animal model of IL-17A overexpression, we discovered that IL-17A increases the level of TNF-α in the brain through the TLR4/NF-κB signalling pathway, aggravates neuroinflammation, and thus exacerbates cognitive impairment. The BV2 cell line is a type of microglial cell line derived from C57/BL6 mice. BV2 cells were used as model cells to further verify the role of IL-17A in neuroinflammation at the cellular level. BV2 cells were stimulated with lipopolysaccharide (LPS) (100 ng/mL) for 0, 4, 8, and 24 h. The culture medium was collected, and the TNF-α level in the culture medium was measured by ELISA. The results showed that the level of TNF-α in the culture medium was significantly higher than that in the control group after 24 h of stimulation (Fig. 4A). BV2 cells were collected, and the expression level of TNF-α mRNA was measured by RT‒qPCR. The results showed that the TNF-α mRNA level in BV2 cells markedly increased after stimulation with LPS for 4 h (Fig. 4B). These results suggest that microglial secretion of TNF-α considerably increases under inflammatory conditions.

We further explored the effect of IL-17A on TNF-α secretion by BV2 cells. The ELISA results showed that the TNF-α levels in the culture medium of BV2 cells that were incubated with IL-17A (20 ng/ml) and LPS for 24 h was considerably higher than those of cells that were incubated with LPS and IL-17A alone (Fig. 4C). The RT‒qPCR results showed that the expression level of TNF-α mRNA in BV2 cells was markedly higher than that in the LPS and IL-17A alone groups after 4 h of coincubation with IL-17A and LPS (Fig. 4D). These results indicate that IL-17A further promotes the synthesis and secretion of TNF-α in microglia during neuroinflammation. In addition, WB or RT‒qPCR were used to measure the expression of IL-17RA, IL17RC, CD80, CD86, MHCII and related proteins in the TLR4/NF-κB signalling pathway. We observed that IL-17A readily upregulated the levels of TLR4, NF-κB, and MYD88 transcripts and stimulated the production of TLR4, NF-κB, MYD88, IL-17RA, IL17RC, CD80, CD86 and MHCII proteins in vitro (Fig. 4E–H, Fig. S3), further confirming that IL-17A increases the TNF-α levels by activating the TLR4/NF-κB signalling pathway.

### IL-17A decreased the activity and phagocytosis of BV2 cells

**Fig. 5:**
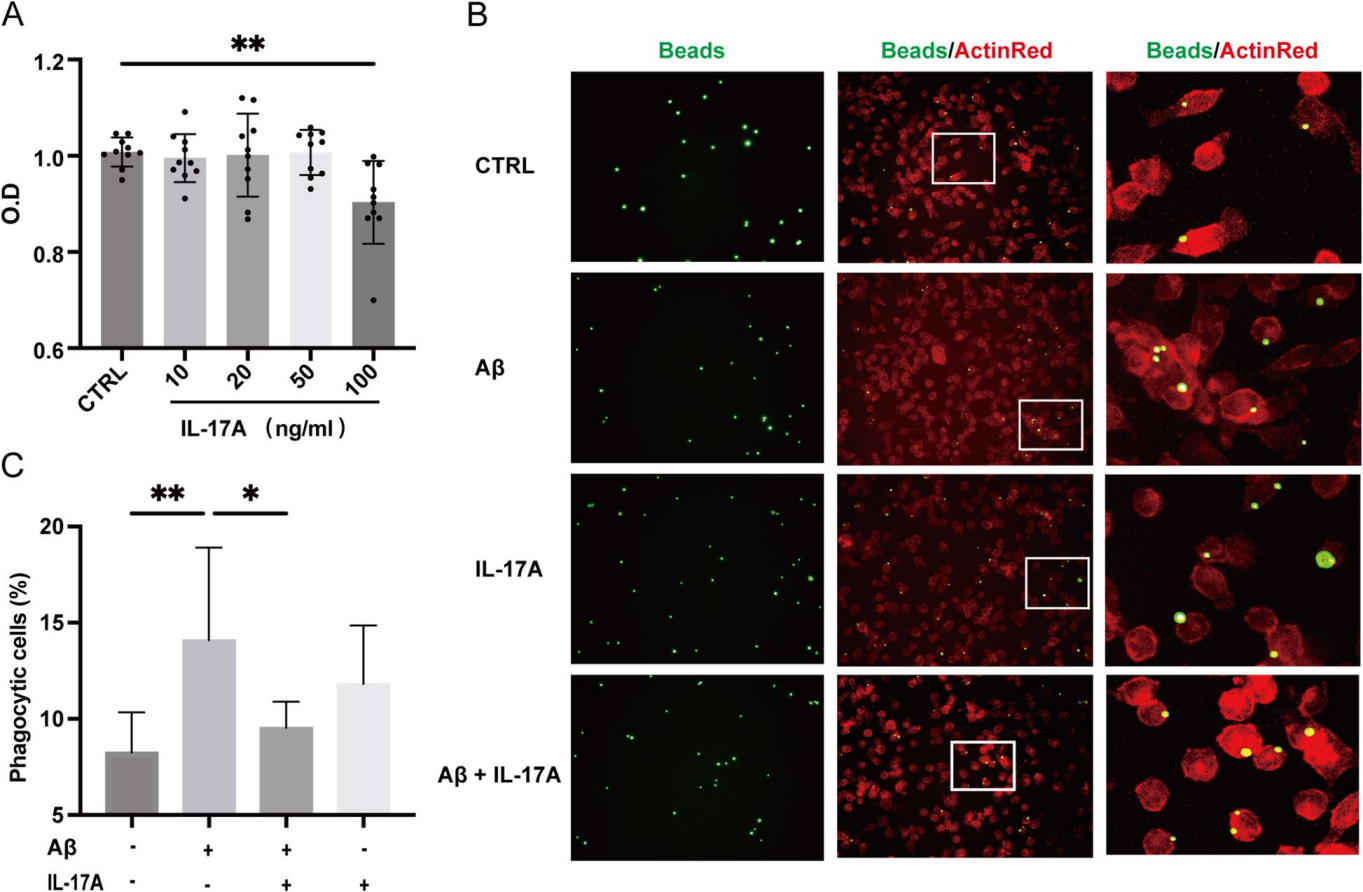
Effect of IL-17A on the activity and phagocytosis of BV2 cells. **A** Effect of IL-17A concentration on BV2 cell activity as shown by the CCK8 method. **B** Phagocytosis efficiency of BV2 cells incubated with IL-17A as determined with a fluorescent phagocytosis assay with latex beads. **C** Quantitative analysis of the number of latex beads that were phagocytosed by BV2 cells that were treated with Aβ and/or IL-17A. The values are expressed as the mean ± standard deviation (****P < 0.0001, ***P < 0.001, **P < 0.01, and *P < 0.05). The data are representative of at least three independent experiments

Through the above studies, we demonstrated that at the cellular level, IL-17A may increase TNF-α secretion by microglia via the TLR4/NF-κB signalling pathway. We then aimed to elucidate the effect of IL-17A on microglial function. First, the cytotoxicity of various concentrations of IL-17A (10–100 ng/ml) towards BV2 cells was determined by Cell Counting Kit-8 (CCK8) assay. Compared with the controls, when the concentration of IL-17A reached 100 ng/ml, the activity of BV2 cells markedly decreased (Fig. 5A), which indicated that high-dose recombinant IL-17A in mice can reduce the activity of BV2 cells. Thus, a high level of IL-17A in the brain tissue may reduce the biological function of microglia and accelerate their apoptosis. Given that activated microglia proliferate, accumulate around Aβ plaques, and respond to neuroinflammation via phagocytosis, we further analysed the morphological changes of activated BV2 cells using a phagocytosis assay. Fluorescence analysis showed that compared with BV2 cells that were treated with Aβ alone, the number of latex beads phagocytosed by BV2 cells that were treated with IL-17A was decreased (Fig. 5B), which indicates that IL-17A weakened microglial phagocytosis. These results suggest that IL-17A may accelerate the deposition of Aβ in the brain tissues of AD model mice or patients by decreasing the phagocytosis of microglia.

### IL-17A promoted Aβ deposition in HT22 cells

**Fig. 6:**
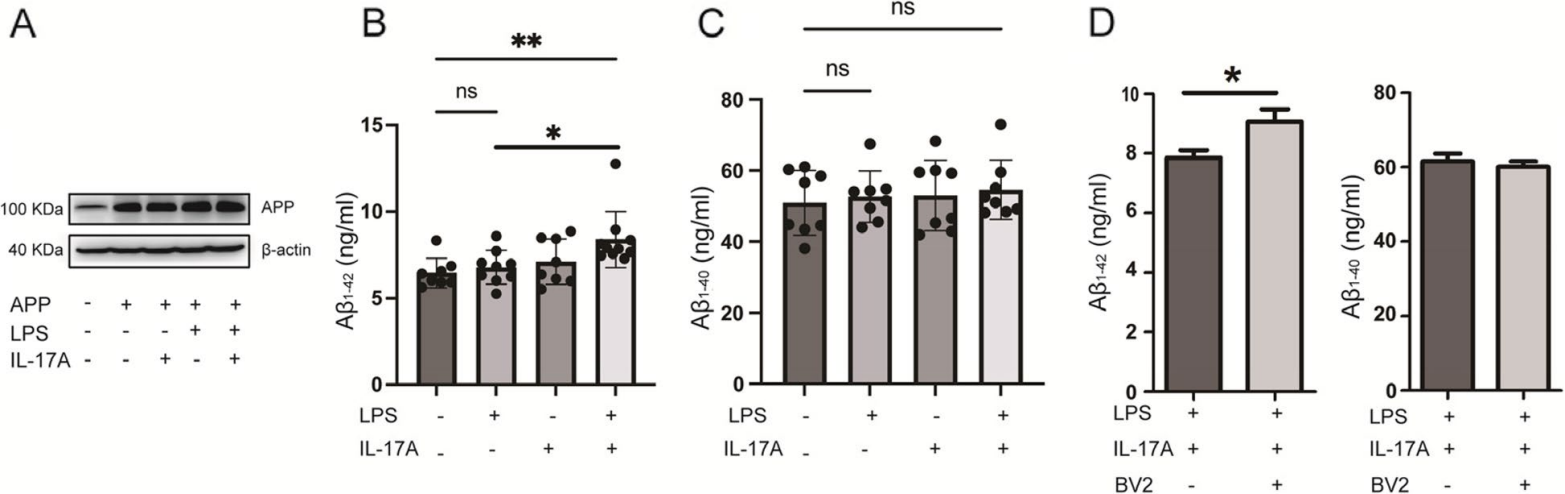
IL-17A promoted Aβ generation in HT22 cells. **A** Expression of APP proteins in HT22 cells that were transfected with APP overexpression plasmid and subsequently incubated with LPS and/or IL-17A for 24 h. **B** Aβ_1-42_ levels in the culture medium of HT22 cells that were transfected with APP-overexpressing plasmids and subsequently incubated with LPS and/or IL-17A for 24 h. **C** Aβ_1-40_ levels in the culture medium of HT22 cells that were transfected with APP-overexpressing plasmids and subsequently incubated with LPS and/or IL-17A for 24 h. **D** Aβ_1-42_ and Aβ_1-40_ levels in the culture medium of HT22 cells that were transfected with APP-overexpressing plasmids and subsequently incubated with LPS and IL-17A or BV2 for 24 h. The values are expressed as the mean ± standard deviation (****P < 0.0001, ***P < 0.001, **P < 0.01, and *P < 0.05). The data are representative of at least three independent experiments

IL-17A stimulates microglia to secrete the proinflammatory factor TNF-α, which exacerbates neuroinflammation. Another important pathological change in AD results from the deposition of Aβ plaques. To examine the effect of IL-17A on APP metabolism, we transfected HT22 cells with the mouse APP plasmid. The expression of the APP protein was markedly upregulated in APP-transfected cells compared with the control, which indicates that the recombinant plasmid was successfully constructed (Fig. 6A). Compared with the control, LPS alone, and IL-17A alone groups, the levels of Aβ_1-42_ in the HT22-APP-transfected cell culture medium of the LPS and IL-17A coincubated groups was significantly increased (Fig. 6B). However, no marked change was observed in Aβ_1-40_ (Fig. 6C). A substantial increase in the level of Aβ_1-42_ was observed in HT22-APP-transfected cells that were coincubated with LPS, IL-17A, and BV2 cells compared with those that were coincubated with LPS and IL-17A_,_ but no significant change was observed in the Aβ_1-40_ levels in the cell culture medium (Fig. 6D). These results suggest that IL-17A and microglia strongly accelerate Aβ deposition in AD model cells.

## Discussion

A previous study reported the relationship between AD and the immune system; the researchers systematically expounded that activated microglia play an important role in neurodegenerative diseases [18]. Since then, the role of neuroinflammation, with microglia as the main research object, in the pathogenesis of AD has attracted increasing attention.

With the progression of AD, the levels of proinflammatory and anti-inflammatory factors in the brain constantly change, which aggravates neuroinflammation. The levels of TNF-α are enhanced in the blood and CSF of AD patients [19] Neuroinflammation has long been considered a key factor in the progression to AD, and TNF-α acts as a key pro-inflammatory factor, which has been found in previous studies to be elevated in cerebrospinal fluid and blood in patients at different stages of AD [20], consistent with the findings in this study in animal models of AD. MCI patients with high levels of TNF-α are more likely to progress to DAT [21]. In the inflammatory state, TNF-α produced by peripheral blood immune cells can enter the brain through the blood–brain barrier. TNF-α has been found in animal models to promote neuronal death by activation of microglia, exacerbating dementia [22]. In our study, the levels of inflammatory cytokines in the brain tissues of APP/PS1 mice were measured, and the mRNA levels of TNF-α in the brain tissues of mice increased. Thus, the increased level of TNF-α in AD brain tissues may be one of the main causes of chronic neuroinflammation.

Many immune cells have been shown to contribute to the production of IL-17A that is observed in mang diseases. In addition, IL-17A triggers other inflammatory factors, such as IL-6, TNF-α, and IL-1β. IL-17A can increase the immunoreactivity and release of inflammatory mediators [23]. In diseases that affect the CNS, the level of IL-17A increases in the CSF and peripheral blood in multiple sclerosis [24]. A previous study showed that the level of IL-17A was elevated in AD model mice. How does IL-17A affect the synthesis and release of inflammatory cytokines in microglia under inflammatory conditions? Our study showed that IL-17A can promote the production of TNF-α in microglia and exacerbate neuroinflammation. The results suggest that IL-17A may affect the pathological process of AD by promoting the secretion of TNF-α by microglia.

To systematically evaluate the relationship between IL-17A and cognitive function, in this study, we intraperitoneally injected APP/PS1 mice with a recombinant IL-17A protein. The results showed that after the intraperitoneal injection of recombinant IL-17A, the mice exhibited prolonged escape latency in the water maze experiment. In addition, immunofluorescence showed that Aβ plaques were substantially increased in the brain, and the number of IBA-1^+^ microglia was also increased, which indicates that IL-17A may exacerbate the progression of AD. In particular, after the intraperitoneal injection of anti-IL-17A neutralizing antibodies, the cognitive function of APP/PS1 mice improved, and Aβ plaques in the brain were reduced. This study further confirmed the important role of IL-17A in the pathogenesis of AD and suggested that IL-17A can be an effective target for the treatment of AD.TLR4 is a membrane protein receptor that is expressed in microglia. When expressed on the cellular membrane, TLR4 exists as a complex with the coreceptor MYD88 to form MyD88-TLR4 complexes, which results in the activation of downstream mediators, including the transcription factor NF-κB, and regulates the expression of proinflammatory molecules, such as TNF-α, IL-1β, and IL-6 [15]. In our study, the effects of IL-17A on the TLR4/NF-κB signalling pathway in BV2 cells were investigated. The results showed that the protein expression levels of TLR4, NF-κB, and MYD88 increased after the addition of IL-17A, which indicates that IL-17A may upregulate the expression of TNF-α by regulating the above signalling pathway.

In our study, LPS-stimulated BV2 cells were used to establish an inflammatory cell model, and the levels of cytokines in the culture medium were measured. When microglia are in an inflammatory state, the secretion of TNF-α by microglia increases, which indicates that microglia perform a neuroinflammatory function via TNF-α. Thus, TNF-α plays an important role in the process of AD. Although the brain was once perceived as an immune-privileged area, researchers have observed the breakdown of the BBB in AD patients [25]. The enhanced permeability of the BBB allows the entry of inflammatory factors into the brain.

Microglia, which are the main innate immune cells of the CNS, originate from erythromyeloid progenitor cells in the embryonic yolk sac. In the brain tissues of AD patients, microglia are distributed near Aβ plaques. Microglia can clear Aβ plaques through their phagocytic function. As the disease progresses, microglia release neurotoxic proteases and inflammatory factors that damage neurons, further exacerbating memory loss [26]. In this study, the CCK8 assay was used to assess the effect of IL-17A on the activity of BV2 cells, and the results showed that a low concentration of IL-17A had no effect on the activity of BV2 cells. In addition, after stimulation of BV2 cells with Aβ_1-42_ to establish a cell model of AD, the addition of IL-17A attenuated the phagocytic ability of the cells. The results showed that when circulatory IL-17A entered brain tissue, microglial activity was not affected but the phagocytosis of microglia was affected. These results suggest that with the progression of AD, the increase in IL-17A levels in the brain leads to the decreased phagocytic ability of microglia, weakening of Aβ plaque clearance ability, and exacerbation of Aβ plaque deposition and disease progression.

The accumulation of Aβ peptides and neurofibrillary tangles are considered the main major hallmarks of AD. Moreover, neuroinflammation has become the third hallmark of the disease. Recent studies have shown that risk factors, such as systemic inflammation, obesity, and traumatic brain injury, may be associated with disease progression [27]. In this study, the secretion of APP amyloid degradation pathway products Aβ_1-42_ increased after IL-17A was added to APP-transfected HT22 cells, which indicates that IL-17A can promote the production of Aβ peptides and exacerbate the process of AD by promoting the APP amyloid pathway.

## Conclusions

**Fig. 7:**
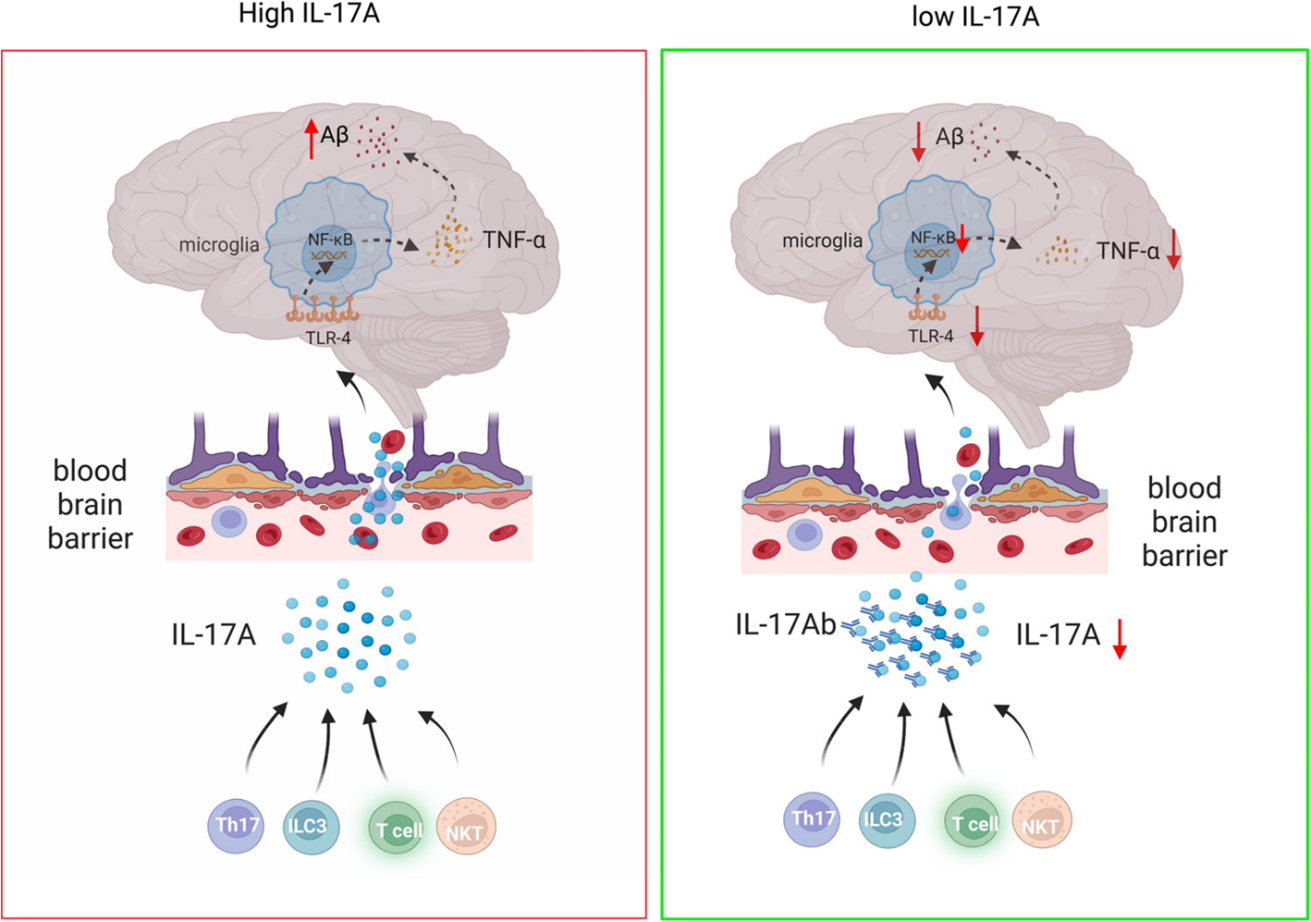
Diagram showing the regulatory mechanism of IL-17A in AD. The level of the cytokine IL-17A that was secreted by peripheral immune cells increased, and the BBB was damaged. Peripheral IL-17A may promote the production of TNF-α through the TLR4/NF-κB signalling pathway, leading to an increase in Aβ production, reducing the phagocytic ability of microglia, weakening the clearance of Aβ, and exacerbating the progression of AD

Our analysis revealed the synergistic effect of IL-17A and TNF-α. IL-17A can promote the secretion of the inflammatory factor TNF-α by microglia and the formation of Aβ plaques and attenuate the phagocytic ability of microglia. However, inhibition of IL-17A can reduce Aβ plaque deposition in the brains of AD patients, decrease neuroinflammation, and slow disease progression (Fig. 7).

### Supplementary Information

**Additional file 1.** 

## Data Availability

The datasets used and/or analysed during the current study are available from the corresponding author on reasonable request.

## Abbreviations

AD: Alzheimer’s disease
TNF-α: Tumor necrosis factor-α
TLR4: Toll-like receptor 4
NF-κB: Nuclear factor-κB
Aβ: β-Amyloid
CNS: Central nervous system
IL-17A: Interleukin-17A
BBB: Blood–brain barrier
CSF: Cerebrospinal fluid
WT: Wild-type
MWM: Morris water maze
MMSE: Mini-Mental State Examination
MoCA: Montreal Cognitive Assessment
HC: Healthy controls
MCI: Mild cognitive impairment
DAT: Dementia of Alzheimer type
VaD: Vascular dementia
PD: Parkinson’s disease
LPS: Lipopolysaccharide
