# Supplementary material for: IL-17A promotes the progression of Alzheimer’s disease in APP/PS1 mice
Source: Immun Ageing. 2023 Dec 14;20:74. doi: 10.1186/s12979-023-00397-x (PMC10720112; doi:10.1186/s12979-023-00397-x)
Supplement: Supplementary file 1 — Additional file 1. [file 12979_2023_397_MOESM1_ESM.zip › Addfile1.docx]

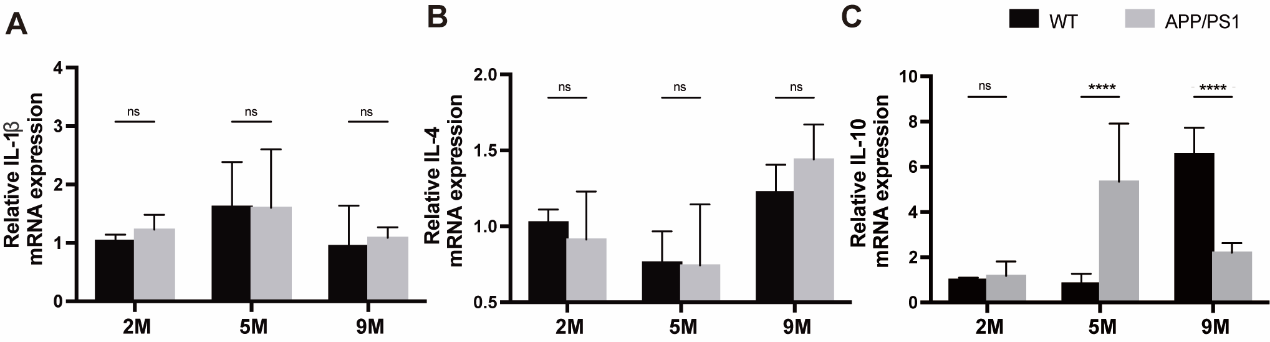


SUPPLEMENTAL FIGURE 1| Studies of the levels of cytokines in APP/PS1 mice of different ages (months). (A) IL-1β mRNA levels in the hippocampus of 2-, 5-, and 9-month-old APP/PS1 mice. (B) IL-4 mRNA levels in the hippocampus of 2-, 5-, and 9-month-old APP/PS1 mice. (C) IL-10 mRNA levels in the hippocampus of 2-, 5-, and 9-month-old APP/PS1 mice. The values are expressed as the mean ± standard deviation (****P < 0.0001). n = 5 for each group. The data are representative of at least three independent experiments.
