# Supplementary material for: IL-17A promotes the progression of Alzheimer’s disease in APP/PS1 mice
Source: Immun Ageing. 2023 Dec 14;20:74. doi: 10.1186/s12979-023-00397-x (PMC10720112; doi:10.1186/s12979-023-00397-x)
Supplement: Supplementary file 1 — Additional file 1. [file 12979_2023_397_MOESM1_ESM.zip › SUPPLEMENTAL FIGURE 3_ESM.docx]

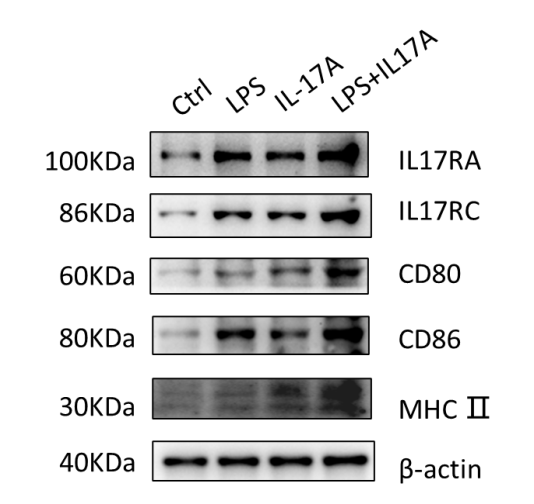


SUPPLEMENTAL FIGURE 3| Expression level of the IL17RA, IL17RC, CD80, CD86 and MHCII after IL-17A stimulation of BV2 cells for 24 h under inflammatory conditions. Data are representative of at least three independent experiments.
