# Supplementary figures and images for: IL-17A promotes the progression of Alzheimer’s disease in APP/PS1 mice
Source: Immun Ageing. 2023 Dec 14;20:74. doi: 10.1186/s12979-023-00397-x (PMC10720112; doi:10.1186/s12979-023-00397-x)

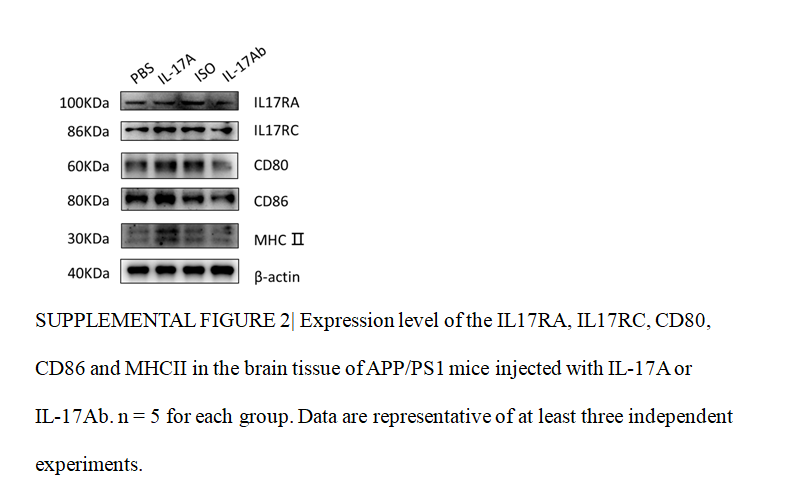

Supplement: Supplementary file 1 — Additional file 1. [file 12979_2023_397_MOESM1_ESM.zip › Addfile 2.png]

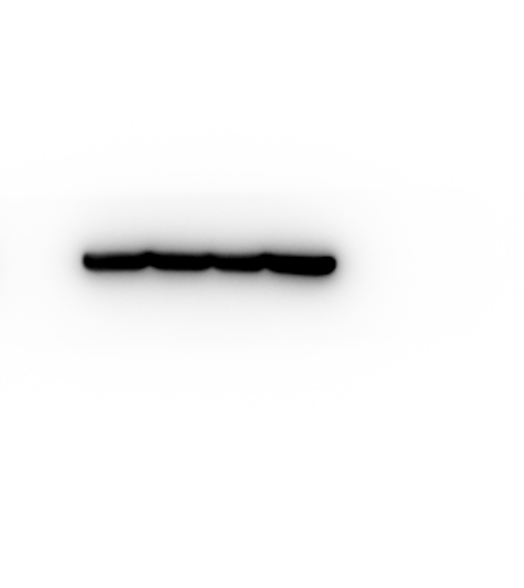

Supplement: Supplementary file 1 — Additional file 1. [file 12979_2023_397_MOESM1_ESM.zip › Fig 3C-a--actin-1.tif]

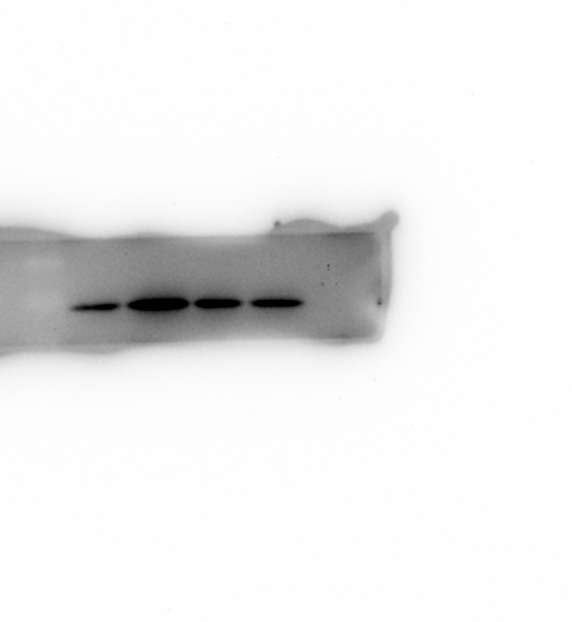

Supplement: Supplementary file 1 — Additional file 1. [file 12979_2023_397_MOESM1_ESM.zip › Fig 3C-MyD88-1.tif]

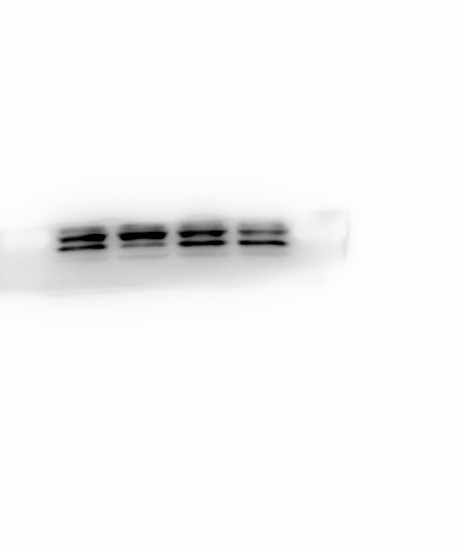

Supplement: Supplementary file 1 — Additional file 1. [file 12979_2023_397_MOESM1_ESM.zip › Fig 3C-NFkB-1.tif]

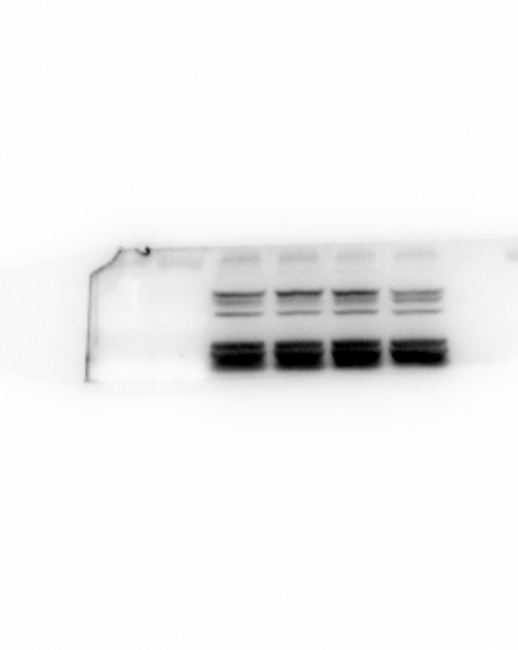

Supplement: Supplementary file 1 — Additional file 1. [file 12979_2023_397_MOESM1_ESM.zip › Fig 3C-TLR4-1.tif]

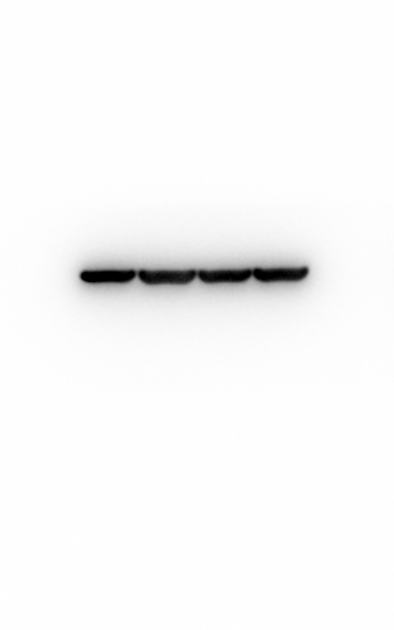

Supplement: Supplementary file 1 — Additional file 1. [file 12979_2023_397_MOESM1_ESM.zip › Fig 4E-a--actin-1.tif]

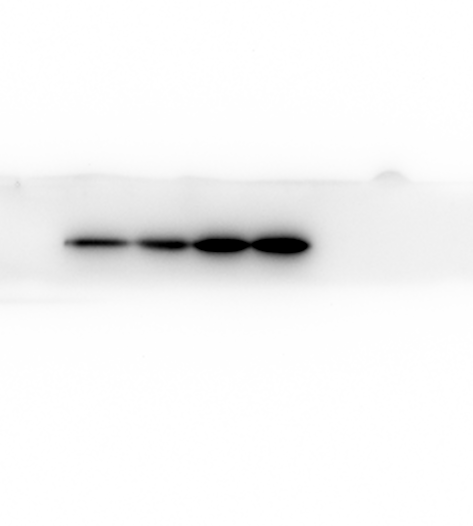

Supplement: Supplementary file 1 — Additional file 1. [file 12979_2023_397_MOESM1_ESM.zip › Fig 4E-MyD88-1.tif]

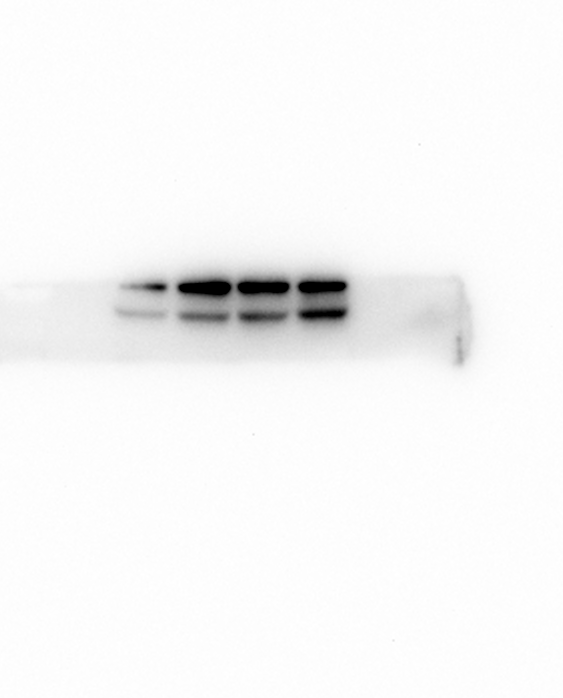

Supplement: Supplementary file 1 — Additional file 1. [file 12979_2023_397_MOESM1_ESM.zip › Fig 4E-NFkB-1.tif]

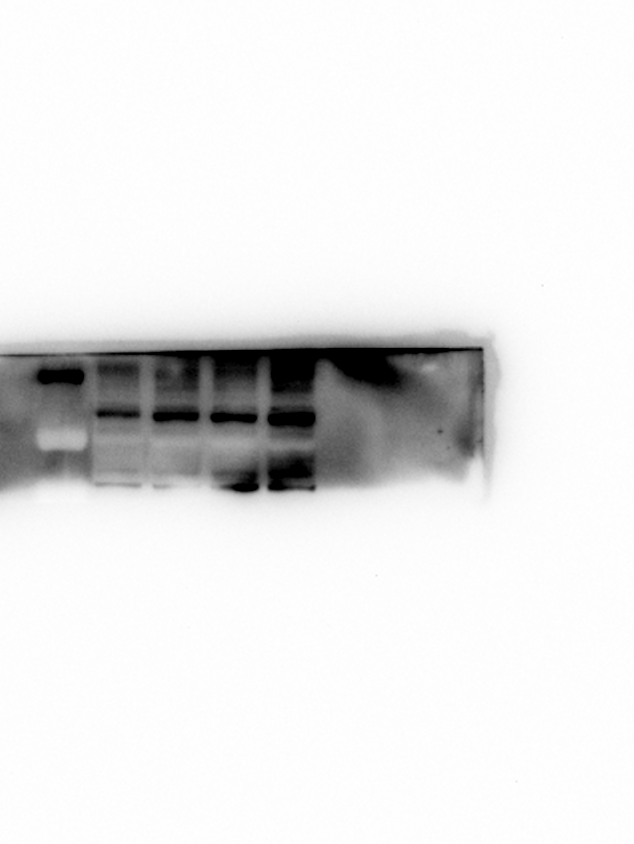

Supplement: Supplementary file 1 — Additional file 1. [file 12979_2023_397_MOESM1_ESM.zip › Fig 4E-TLR4-1.tif]

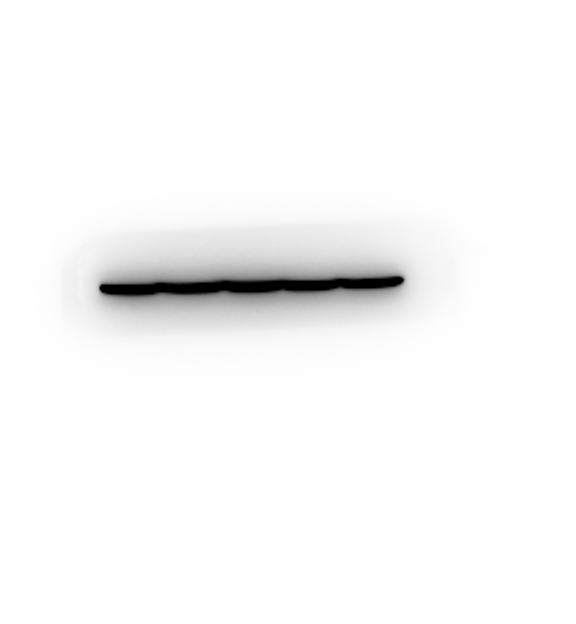

Supplement: Supplementary file 1 — Additional file 1. [file 12979_2023_397_MOESM1_ESM.zip › Fig 6A-a--actin-1.tif]

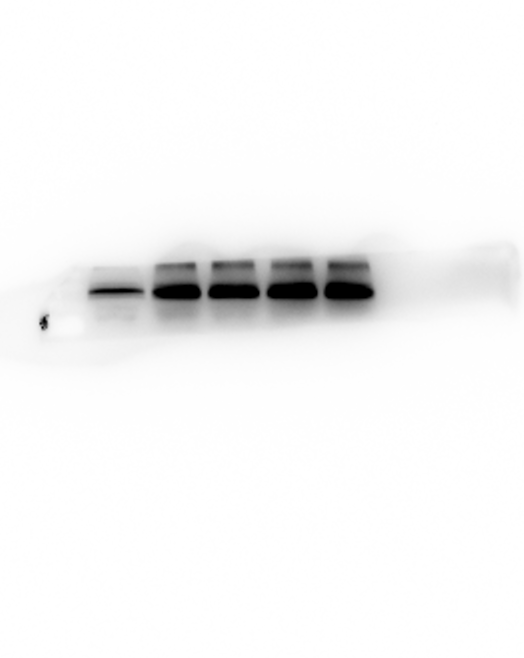

Supplement: Supplementary file 1 — Additional file 1. [file 12979_2023_397_MOESM1_ESM.zip › Fig 6A-app-1.tif]
